# Synthesis, Spectral and Thermal Studies of New Rutin Vanadyl Complexes

**DOI:** 10.3390/molecules15031578

**Published:** 2010-03-10

**Authors:** Valentina Uivarosi, Stefania Felicia Barbuceanu, Victoria Aldea, Corina-Cristina Arama, Mihaela Badea, Rodica Olar, Dana Marinescu

**Affiliations:** 1 Faculty of Pharmacy, Carol Davila University of Medicine and Pharmacy, 6 Traian Vuia St, 020956 Bucharest, Romania; E-Mails: stefaniafelicia_barbuceanu@yahoo.com (S.F.B.); victoria.aldea@yahoo.com (V.A.); corinaarama@yahoo.com (C.-C.A.); 2 Faculty of Chemistry, University of Bucharest, 90-92 Panduri St, 050663, Bucharest, Romania; E-Mails: e_m_badea@yahoo.com (M.B.); rodica_m_olar@yahoo.com (R.O.); marinescu@unibuc.ro (D.M.)

**Keywords:** flavonoid, rutin, vanadyl complex, spectroscopic techniques, thermal analysis

## Abstract

Complexes between oxovanadium (IV) cation and flavonoid derivatives were developed recently in order to increase the intestinal absorption and to reduce the toxicity of vanadium compounds. For these reasons, is interesting to investigate the complexation process between flavonoid rutin (Rut) and vanadyl cation in order to isolate new complexes. Two new complexes [VO(Rut)(H_2_O)_2_](SO_4_)_0.5_ 2H_2_O and [VO(Rut)_2_] 4H_2_O have been obtained and characterized by elemental and thermal analyses and several spectroscopic techniques (ESI-MS, IR, UV-Vis, fluorescence). The studies concerning complex formation between vanadyl and rutin (Rut) performed in different solutions show the formation of mononuclear complexes with 1:1 and 1:2 metal to ligand stoichiometry.

## 1. Introduction

**Figure 1:**
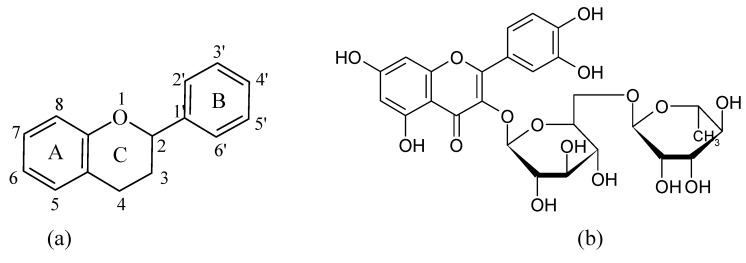
(a) Base structure of flavonoids. (b) Structure of rutin, a flavonol derivative.

Flavonoids belong to a large group of polyphenolic phytochemicals present in seeds, fruit skin, peel, and bark of plants and they display several biological effects like antibacterial [1], antiviral, anti-inflammatory [2,3], antiallergic [4], antithrombotic [5] antimutagenic, antineoplastic [6], as well as neuroprotective properties [7]. Part of these actions can be explained by their antioxidant properties exerted through direct free radical scavenging [8,9] The major flavonoid classes are flavones, flavanonols, flavonols, flavanones and isoflavones. The flavonoid nucleus (Figure 1) consists of benzo-γ-pyrone (ring A and ring C) and benzene (ring B) moieties with hydroxyl, carbonyl, sugar or methyl groups are attached to this base structure.

Flavonoids are good chelating ligands and there are three domains that can interact with metal ions: the 3’,4’-dyhydroxy group located on the B ring, the 3-hydroxy or 5-hydroxy and the 4-carbonyl groups in the C ring. A number of recent studies have suggested that the chelating properties of flavonoids can be assigned to the presence of the 3- or 5-hydroxypyran-4-one, rather than the *ortho*-hydroxyl groups in the B ring [10].

In addition to direct reaction with free radicals, chelation of metal ions involved in the production of reactive oxygen species is thought to be another mechanism of flavonoids’ antioxidant activity [11]. Moreover, the flavonoid chelated compounds are more effective free radical scavengers than flavonoids alone [12,13,14]. It was also observed that some flavonoids possess insulin-mimetic activity based on their anti-hyperglycemic effect [15,16]. These properties are increased upon complexation with oxovanadium(IV) ion [17,18,19] and as a result a series of oxovanadium(IV) complexes with various flavonoid ligands has been investigated as potential anti-diabetes drugs as well as for their antitumor and osteogenic activities [20,21].

Among the flavonoids, rutin (3,3’,4’,5,7-pentahydroxyflavone-3-rhamnoglucoside, quercetin-3-*O*-rutinose, vitamin P, Figure 1) is a bioderivative having a flavonol structure. Rutin is a powerful antioxidant [22] with anti-inflammatory activity [23,24], and exerts renal-protective effects on the ischemia/reperfusion induced renal injury [25], ethanol-induced gastric injury [26], and DNA damage induced by mitomycin [27]. Rutin has also anticonvulsivant effects in the brain [28]. A series of complexes of this ligand display an enhancement of free radical scavenger ability [29,30] and of the anti-inflammatory activity [31]. So far, the interactions of rutin with oxovandium (IV) ion were only studied for analytical purposes [32]. The aim of this work was to isolate some new vanadyl ion and rutin (Rut) ligand compounds. These complexes, [VO(Rut)(H_2_O)_2_](SO_4_)_0.5_ 2H_2_O (**1**) and [VO(Rut)_2_] 4H_2_O (**2**), were characterized by elemental and thermal analyses as well as a series of combined spectroscopic techniques (ESI-MS, IR, UV-Vis and fluorescence). Moreover, study of complexation process between rutin and vanadyl ion in different solutions was performed in order to optimize the synthesis methods for the novel oxovanadium (IV) complexes with potential insulin-mimetic activity.

## 2. Results and Discussion

### 2.1. Characterization of the complexes

**Figure 2:**
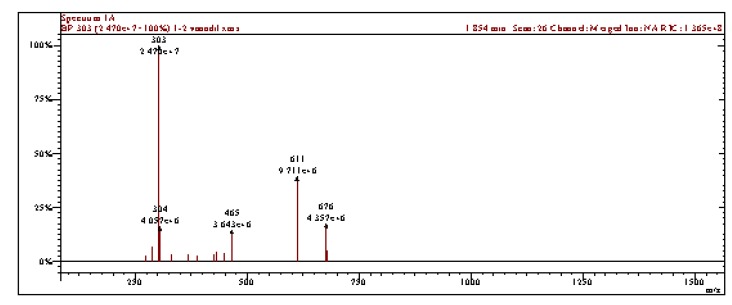
Mass spectrum of [VO(rutin)(H_2_O)_2_](SO_4_)_0.5_ 2H_2_O.

In the mass spectrum of complex **1** the peak at *m/z* 676 corresponds to the [VO(Rut)]^+^ ion (Figure 2). The molecular ion fragmentations lead to different fragments with *m/z* 658 (corresponding to water loss), *m/z* 530 (rhamnose loss), *m/z* 514 (ruthoside loss), *m/z* 368 (quercetin-vanadyl complex), *m/z* 303 (quercetin), while other peaks correspond to various glucose fragments. In the mass spectrum of complex **2** the peaks at *m/z* 1,284 and 676 corresponding to [VO(Rut)_2_]^+^ and [VO(Rut)]^+^ ions are present. These results are consistent with a metal to ligand as stoichiometry of 1:1 and 1:2, respectively for these complexes.

The IR spectra of the complexes (Table 1) exhibit the characteristic patterns of rutin that generate bands about 3,430 [ν(OH)], 1,656 [ν(C=O)] and 1,296 cm^-1^ [ν(C-O-C)].

**Table 1:** Selected absorption maxima (cm^-1^) for rutin and complexes.

Compound	ν(OH)	ν(C=O)	ν(C-O-C)	ν(V=O)	ν_3_(SO_4_^2-^)	ν_4_(SO_4_^2-^)
Rutin	3,429	1,656 (s)^*^	1,296 (s)			
**1**	3,401	1,631 (s)	1,296 (s)	983 (m)^**^	1,023 (s)	609 (m)
**2**	3,422	1,622 (s)	1,294 (s)	972 (m)		

^*^ s: strong, ^**^ m: medium.

The band assigned to the carbonyl group is shifted to a lower wavelength comparing with that of the free ligand, proving its coordination. Supplementary bands around 980 cm^-1^ for both complexes are assigned to the ν(V=O) stretching mode. The absorptions of the sulfate in the spectrum of **1** at 1,023 cm^-1^ for ν_3_ and 609 cm^-1^ for ν_4_, respectively, demonstrate that it acts as a free ion [33]. The IR data suggest that the favored metal site is the 5-hydroxypyran-4-one moiety rather than the *ortho*-hydroxyl groups in the B ring.

**Figure 3:**
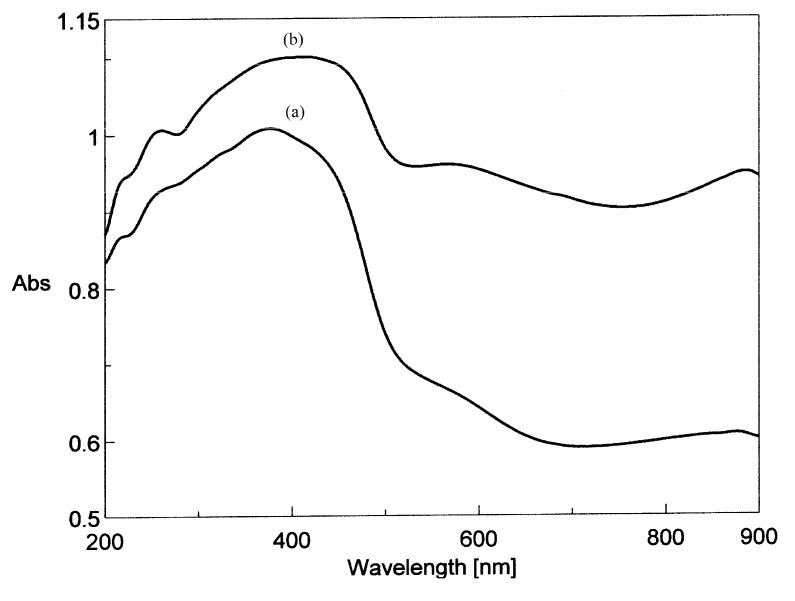
Diffuse reflectance electronic spectra of (a) [VO(rutin)(H_2_O)_2_](SO_4_)_0.5_ 2H_2_O and (b) [VO(Rut)_2_] 4H_2_O.

The diffuse reflectance electronic spectra of complexes in the visible region (Table 2) show the characteristic bands of VO^2+^ in a square pyramidal environment (Figure 3). The absorptions at around 880 nm and the shoulders at about 570 are assigned to the spin allowed ^2^B_2_ → ^2^E, and ^2^B_2_ → ^2^B_1_ transitions [34]. The shoulder at about 420 nm can be assigned to the ligand-to-metal charge transfer (LMCT) band.

**Table 2:** Absorption maxima from UV-Vis spectra.

Compound	λ (nm)				
Rutin	260	360	-	-	-
(**1**)	261	377	420 (sh)	579 (sh)^*^	879
(**2**)	260	382	417	566	886

^*^ sh: shoulder.

In the UV region of spectrum two major absorption maxima are also typically observed for the flavonoid structure. The first absorption maximum, observed at 260 nm (band II) can be considered as originating from π-π* transitions in the A ring, a benzene system, and the second absorption maximum, observed around 370 nm, may be assigned to transitions in the B ring, a cinnamoyl system. This band is broad, as a result of overlapping with LMCT band and after coordination is shifted to higher wavelength according to literature data [35].

**Figure 4:**
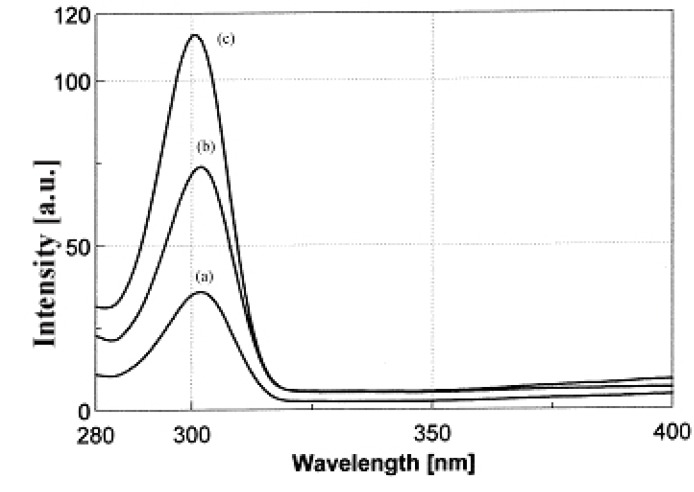
Fluorescence spectra of (a) rutin trihydrate, (b) [VO(rutin)(H_2_O)_2_](SO_4_)_0.5_ 2H_2_O and (c) [VO(Rut)_2_] 4H_2_O (λ_ex_ = 260 nm, λ_em_ = 302 nm).

The fluorescence analysis shows that rutin itself exhibits a strong fluorescence, but the fluorescence intensity of the complexes are stronger than those of the ligand (Figure 4) under the same conditions (λ_ex_ = 260 nm, λ_em_ = 302 nm). The higher fluorescence intensities of the complexes related to the free rutin, are assigned to the coordination of the ligand to the oxovandium (IV) ion. The coordination increases the rigidity of the ligand structure and increases the fluorescence quantum yield by reducing the probability of non-radiative dissipation process.

Thermal analysis was performed in order to establish the number and the nature of water molecules and to elucidate the stoichiometry and composition of the complexes. The results are presented in Table 3.

**Table 3:** Thermal behavior data (in synthetic air) for complexes.

Complex	Step	Thermal effect	Temperature range/°C	Δm_exp/_%			Δm_calcd/_%		
[VO(rutin)(H_2_O)_2_](SO_4_)_0.5_ 2H_2_O	1	Endothermic	78–140		9.04		9.04	
	2	Exothermic	140–388		40.52		40.66	
	3	Exothermic	388–812		33.94		33.88	
	4	Exothermic	812–900		4.98		5.02	
[VO(Rut)_2_] 4H_2_O	1	Endothermic	55–94		5.38		5.31	
	2	Exothermic	94–362		47.68		47.75	
	3	Exothermic	362–900		40.87		40.83	

**Figure 5:**
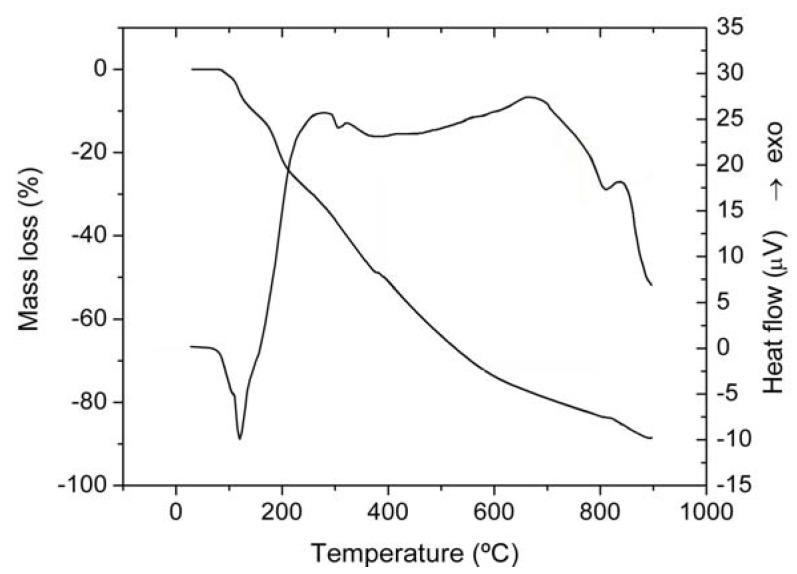
TG and DTA curves of [VO(rutin)(H_2_O)_2_](SO_4_)_0.5_ 2H_2_O.

Complex **1** decomposes in four steps up to 900 °C (Figure 5). The elimination of four water molecules occurs between 78–140 °C in the first step (mass loss found/calcd.: 9.04/9.03%). This endothermic step is not a single one, as the four water molecules seems to be released in two relatively equal stages as both the TG and DTA curves indicate. This is in concordance with the proposed formula of the complex that contains both coordinated and crystallisation water. The next two steps correspond to the oxidative degradation of organic ligand while the sulphate ion decomposition occurs as final step with V_2_O_5_ formation.

**Figure 6:**
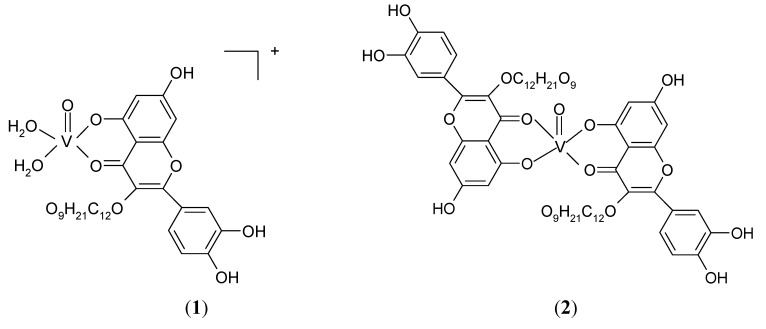
The proposed coordination for [VO(Rut)(H_2_O)_2_]^+^ (**1**) and [VO(Rut)_2_] (**2**).

For complex **2** the water molecules are released up to 94 °C, indicating their crystallisation nature (mass loss found/calcd.: 5.38/5.31%). This endothermic step is also followed by oxidative degradation of the organic ligand that occurs in two distinct steps with V_2_O_5_ as final residue. On the basis of the above data the proposed coordination for the complexes is as follows (Figure 6):

### 2.2. Complex formation and stability studies

The influence of the pH was studied by adding 2 mL of buffer solution (pH range 1–9) to 10 mL of an equimolar mixture of rutin and vanadyl sulphate. It was noticed that starting with pH 8 the complex formed was insoluble in the water-methanol mixture. So all other tests were done without any added buffer (pH of the mixture, potentiometrically measured, was found to be 5.7–6.1).

**Figure 7:**
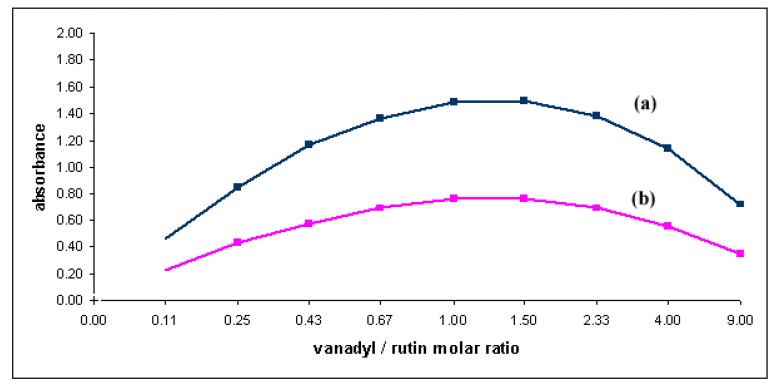
Plot of the VO^2+^-rutin complex formation in a CH_3_OH-H_2_O (70:30) mixture; (a) C_VO2+_ + C _rutin_ = 0.10 mM. (b) C_VO2+_ + C _rutin_ = 0.05 mM.

According to the literature, the most reliable results of the stoichiometric composition of flavonoid complexes were obtained by the method of continual variation of equimolar solutions [36]. Consequently, the stoichiometry of the complex in methanol solution was investigated using both Job’s method [35] (spectra recorded at total constant concentration) and the slope ratio method. It was noticed that the intensity of the absorption at 416 nm was a function of both vanadyl and rutin concentration. When plotting the absorbance at 416 nm versus mole fraction of ligand, the results indicated that 1:1 chelate is formed whenever the solvent was a mixture of water and methanol (Figure 7).

**Figure 8:**
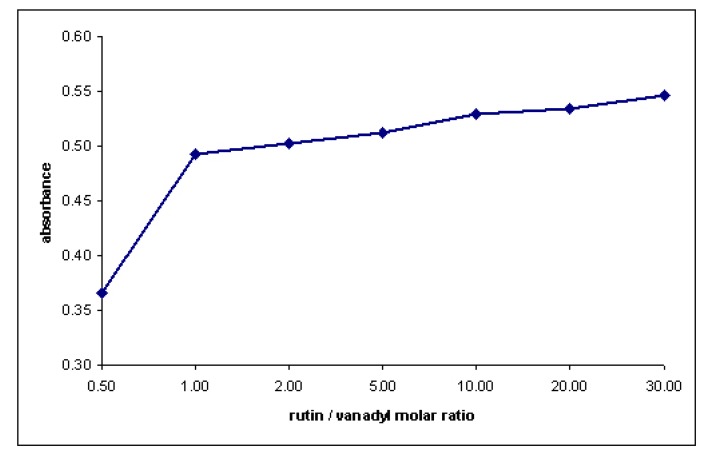
Absorbance versus rutin concentration (C _vanadyl sulfate_ = 0.05 mM).

The results were consistent with data obtained using slope ratio method (the slope ratio was 0.987:1.022 – vanadyl constant excess and rutin constant excess, respectively). To determine the apparent stability constant of the complex, absorption spectra of solutions containing 1:1 to 1:30 were recorded and the absorbance at 416 nm measured (Figure 8).

The dissociation degree α was estimated from the absorbance of the solution when all the vanadyl present is complexed (A_m_) and the absorbance at the stoichiometric molar ratio (A_s_):

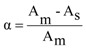
(1)

K was computed from the instability constant K_i_:

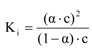
(2)

The average value of the stability constant was calculated as 1.62 × 10^4^ (log β = 4.21).

**Figure 9:**
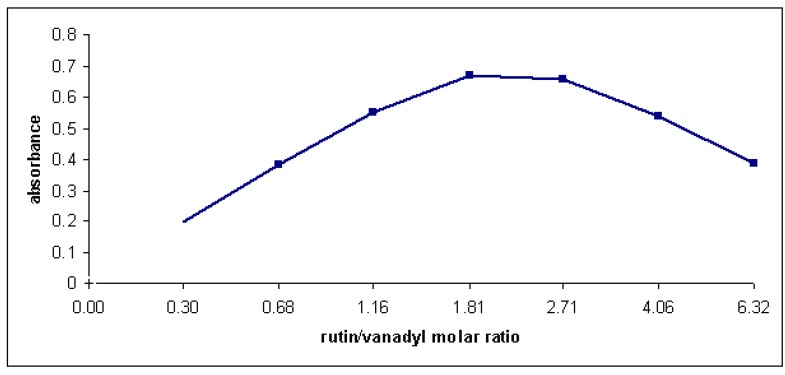
Plot of the VO^2+^-rutin complex formation in aqueous solution (pH 6); C_VO_^2+^ + C_rutin_ = 0.10 mM.

The results indicate that the 1:1 complex is predominant in methanol-containing solutions and this complex is moderately stable. Job’s method has been also performed in order to investigate the stoichiometry of the complex in aqueous solutions at pH 6 (phosphate buffer). Under these conditions a complex with 1:2 metal to ligand ratio was formed (Figure 9).

## 3. Experimental

### 3.1. General

Rutin trihydrate (>90% HPLC chemicals) was purchased from Fluka (Sigma-Aldrich Chemie GmbH Division, Germany), vanadium (IV)-oxide sulfate pentahydrate from Riedel-deHaen (Sigma Aldrich Chemie GmbH Division, Germany), methanol for spectroscopy from Merck (Merck KGaA, Germany), ultrapure water. All reagents were used without further purification. Rutin has very low solubility in water and so solutions of this ligand were prepared using 80:20 vol/vol methanol-water. Mass spectra were recorded by electrospray ionization tandem mass spectrometry (ESI-MS) technique with the following procedure: a sample (1 mg) was dissolved in 1:1 chloroform-methanol and after 30 sec of exposure to an ultrasound bath the solution was injected directly into the electrospray interface of a 1200 L/MS/MS (Varian) mass spectrometer. The injection was performed using a Prostar 240 SDM (Varian) pump with 0.02 mL/min. flow. Air at 200 °C and 19 psi was used for desiccation and nitrogen at 42 psi was used for dispersion. Molecular ions scanning range (m/z) was 150-1500. IR spectra were recorded in KBr pellets with a FT-IR VERTEX 70 (Bruker) spectrometer in the range 400–4000 cm^-1^. Electronic spectra by diffuse reflectance technique, with Spectralon as reference sample, were recorded in the range 200–900 nm, on a Jasco V 650 spectrophotometer. Fluorescence spectra in solid state were recorded on a Jasco FP 6500 spectrofluorometer. The heating curves (TG, and DTA) were recorded using a Labsys 1200 SETARAM thermobalance with a sample weight between 8–12 mg over the temperature range of 20–900 °C and a heating rate of 10 °C/min. The measurements were carried out in synthetic air atmosphere (flow rate 16.66 mL/min) by using alumina crucible. The chemical analyses of the complexes were performed on a Perkin Elmer PE 2400 analyzer (for C, H, N, S) and an AAS Carl Zeiss Jena AAS1 spectrometer (for V).

Solution spectrophotometric studies were carried out using a Perkin Elmer Lambda 2 UV-Vis spectrometer, in 1.0 cm fused silica cells. To determine the composition and apparent stability of the complex in methanol medium, solutions of vanadium (IV) oxide sulphate and rutin in 20/80 (v/v) mixture of water and methanol were mixed just before the determination. The composition of the chelate **1** was determined both using the continual variation method (Job) and the slope ratio method. When using Job’s method, solutions (1 mM) of each component were used in ratios varying from 1:9 to 9:1. The measurements were performed at 416 nm, absorption characteristic for charge transfer band in complex. In the slope ratio method, first the concentration of VO^2+^ was kept constant, 1 mM, and the concentration of rutin was varied between 0.05 mM and 0.1 mM; then rutin concentration was maintained constant, 1 mM, and the concentration of vanadyl sulphate varied in the range 0.05 mM and 0.1 mM. The stability of the complex was estimated using the molar ratio method: the [vanadyl]: [rutin] ratios varied between 1:1 and 1:30. The composition of complex **2** was determined using Job’s method for aqueous solution at pH 6. The pH of solutions was controlled using phosphate buffer (Na_2_HPO_4_/KH_2_PO_4_). Solutions (1 mM) from each component were used in ratios varying from 1:3 to 6:1. The measurements were performed at 367 nm versus individual reference containing rutin in adequate concentrations.

### 3.2. Synthesis of the complex ***1***

Complex **1** was prepared by dissolving 1 mmol of VOSO_4_ 5H_2_O (0.2529 g) in methanol (50 mL) and then 1 mmol of the rutin trihydrate (0.6645 g) was added while stirring. The solution was refluxed for 3 h. After few days the green crystals of the complex grown by a slow evaporation in air were filtered and washed with water. The solid product was dried in air. Calc. for C_27_H_37_O_23_S_0.5_V (796.55): C, 40.7; H, 4.7; S, 2.0; V, 6.4. Found: C, 40.9; H, 4.7; S, 1.8; V, 6.2%.

### 3.3. Synthesis of the complex ***2***

Complex **2** was prepared with the following procedure: 2.00 g rutin trihydrate (3.01 mmol) was dissolved in 50 mL of distilled water containing a few NaOH pellets. To the resulting solution, a saturated solution of VOSO_4_ 5H_2_O (in the molar ratio VO: ligand 1:2) was added dropwise, under continuous stirring. The pH of the solution was adjusted to 6 with 1 M H_2_SO_4_. After a in a few days a green solid precipitated. The sparingly soluble product was filtered off through a fritted glass funnel, washed several times with water and dried in desiccator over CaCl_2_. Calc. for C_54_H_66_O_37_V (1358.03): C, 47.8; H, 4.9; V, 3.7. Found: C, 47.8; H, 4.8; V, 3.6%.

## 4. Conclusions

Two new complexes of vanadyl ion with the flavonoid rutin have been obtained in solid state and characterized. The composition and the probable structure of the complexes have been established by microchemical analyses and several spectroscopic techniques. Mass spectra revealed the principal fragments of the compounds, the IR spectra show that the benzoyl moiety is the basic site for metal chelation, UV-Vis spectra evidenced the square pyramidal geometry of the vanadyl ion, while the fluorescence spectra add supplementary proofs for the coordination process. Thermal analysis revealed the number and the nature of water molecules and offer information about the stoichiometry and composition of the complexes. Spectrophotometric solution studies indicate that in methanol-water solutions only a mononuclear complex with 1:1 metal to ligand stoichiometry with a moderate stability constant can be obtained.
